# Relationship between spiritual well-being with anxiety and depression among cancer patients

**DOI:** 10.1371/journal.pone.0322923

**Published:** 2025-06-18

**Authors:** Mohsen Vakili Sadeghi, Mohammad Ali Hossein Tehrani, Hajar Pasha, Angela Hamidia, Majid Nabipour, Soraya Khafri, Mohammad Hadi Yadollahpour

**Affiliations:** 1 Cellular and Molecular Biology Research Center, Health Research Institute, Babol University of Medical Sciences, Babol, Iran; 2 Cancer Research Center, Health Research Institute, Babol University of Medical Sciences, Babol, Iran; 3 Student Research Committee, Babol University of Medical Sciences, Babol, Iran; 4 Infertility and Reproductive Health Research Center, Health Research Institute, Babol University of Medical Sciences, Babol, Iran; 5 Social Determinants of Health Research Center, Health Research Institute, Babol University of Medical Sciences, Babol, Iran; University of Sao Paulo, School of Medicine, BRAZIL

## Abstract

**Background:**

Spiritual health is one of the basic concepts regarding how to deal with the problems caused by the disease. Anxiety and depression are common psychological consequences that affect the treatment process in cancer patients. Therefore, the present study has been conducted to explore relationship between spiritual well-being with anxiety and depression among cancer patients.

**Methods:**

A total of 200 eligible cancer patients were included in this cross-sectional survey. Questionnaires of the Hospital Anxiety and Depression Scale (HADS) and Spiritual Well-Being Scale (SWB) were provided to patients.

**Results:**

The mean anxiety and depression scores were 9.98 ± 3.74 and 9.68 ± 3.32, respectively. Nearly half of the patients had anxiety and depression disorders. Age (β = -.300, P = .017) was a significant negative predictor for anxiety, and also education (β = -.885, P = .004) was a significant and negative predictor for depression. The mean score of patients’ spiritual well-being was 76.61 ± 20.01, and its dimensions including existential well-being and religious well-being were 37.35 ± 9.78, and 39.27 ± 10.38, respectively. The majority of patients had a moderate level of spiritual well-being (81%). There was a statistically significant relationship between educational levels and spiritual well-being (P = 049), and religious well-being (P = 033). The spiritual well-being could significantly and negatively predict anxiety (β = -0.154, P < 0.001) and depression (β = -.134, P = < 0.001). There was a significant and inverse relationship between religious well-being with anxiety (rho = -.832, P < 0.001) and depression (rho = -.842, P < 0.001), and between existential well-being with anxiety (rho = -.830, P < 0.001) and depression (rho = -0.813, P < 0.001). There was a significant positive relationship between anxiety and depression (rho = 0.717, P < 0.001). The highest percentage of patients with depression disorder had more anxiety (75.6%).

**Conclusions:**

Spirituality can serve as a protective factor for psychological morbidity. Spirituality wellbeing-based care programs are suggested as a good method to promote mental health in cancerous patients.

## Introduction

Cancer is the second cause of death in the world, and is responsible for about 10 million deaths in 2020; it has placed a heavy burden on the health-treatment services systems [1]. In 2020, about 132,000 people were diagnosed with cancer throughout Iran annually, and about 80,000 people died from cancer [2].The death rate of cancer in Iran ranks third after cardiovascular diseases, accidents, and other natural phenomena, and for this reason, it has been one of the research priorities [3]. The most common cancers in the country are breast, prostate, colorectum, stomach, and leukemia [4].

Diagnosing cancer is a very unpleasant and unbelievable experience for every person, which can disrupt the job, socio-economic status, and family life and lead to the destruction of the patient’s life. These effects especially include different aspects of the quality of life and unfortunate psychological consequences, therefore maintenance of mental health must be considered as an essential part of all cancer prevention and treatment [5]. The overall 12-month prevalence for any mental disorder in cancer patients was 39.4%, and that for anxiety disorders was 15.8%. Additionally, the lifetime prevalence for any mental disorder was 56.3%, and that for anxiety disorders was 24.1% [6]. Grassi et al. (2023) showed that the frequency of anxiety and depression was relatively high in cancer patients, and hurt patients’ performance status, quality of life, length of hospital stay, and even the outcome of treatment; therefore the evaluation and treatment of these two disorders are very important in these patients [7].

Depression is one of the mood disorders that are accompanied by a decrease in energy, feelings of guilt, difficulties in concentration, anorexia, and thoughts of death and suicide. While, anxiety is an unpleasant widespread feeling and worry that is often accompanied by one or more physical feelings such as fatigue, nervousness, restlessness, fear, insomnia, tachycardia, irregular and rapid breathing, sweating, headache, dizziness, difficulty in concentration and memory [7,8], depression and death anxiety are common problems in cancer patients that can affect their mental health [8]. The overall prevalence of depression and anxiety in cancer patients in Iran were reported to be 50.1%, 40.9%; respectively [9]. On the other hand, spiritual well-being is one of the factors affecting other aspects of health. This has recently led the World Health Organization (WHO) to state humans as having physical, psychosocial, and spiritual characteristics [10]. The dimension of spirituality, which is in the direction of human growth and development, has also been mentioned as the fourth dimension by WHO [11].

Spiritual well-being is having a sense of acceptance, positive emotions, and a positive mutual relationship with a sovereign and superior holy power, others, and oneself, which is achieved through a dynamic and coordinated cognitive and emotional process. It is related to the concept of self-transcendence and reflects a person’s value system [12]. Spirituality is the central core of all dimensions of existence in humans. Creating and developing a sense of spirituality may be one of the appropriate ways to adapt to diseases [13].

Spiritual well-being has two dimensions, existential well-being and religious well-being. Existential well-being discusses how people adapt to themselves, society, or the environment. Religious well-being focuses on how people perceive health in their spiritual lives when they are connected to a higher power. In other words, existential well-being reflects the relationship of a person with himself, others, and the environment, and religious well-being reflects the relationship with an infinite power [14].

A review of literature also indicated that factors affecting the spiritual well-being of Iranian subjects were classified into five factors including individual factors, knowledge factors, religious factors, cultural factors, and psychological factors. According to the findings, religious factors among the identified factors had the greatest impact on the spiritual well-being [15].

Using spirituality is often a constructive coping strategy for improving people’s psychological health. Spiritual well-being is considered one of the basic concepts about how to face the problems and tension caused by illness. Investigations have shown that it can represent a set of abilities, capacities, and spiritual resources, which increases adaptability and, the mental health of people [16].

There is little information about spiritual well-being and the mental state of cancer patients who are treated with different therapeutic measures, and seems that a comprehensive care program that includes spiritual well-being is more effective in reducing the mental problems of these [17]. Furthermore, anxiety is among the common disorders that affect the treatment process in cancer patients. The existence of paradoxical relationships between spiritual health and anxiety in cancer patients in foreign studies and the lack of similar studies in the country necessitated the design and implementation of a study with the aim of determining relationship between spiritual well-being with anxiety and depression among cancer patients.

## Materials and methods

### Study design and setting

This cross-sectional study was conducted in Ayatollah Rouhani Hospital in Babol City, Mazandaran Province, Iran from November 25, 2019 to September 10, 2020. Ayatollah Rouhani Hospital is a public center belonging to Babol University of Medical Sciences and is one of the most referral public centers in Mazandaran Province for cancerous patients. It accepts patients from all cities in the province. All cancer patients referred to this hospital who met the inclusion criteria and were willing to cooperate were included in this study. Written consent forms were obtained from all participants prior to their entry into the study. For recruitment, the researcher selected eligible subjects based on the criteria in this research through daily referred to the Ayatollah Rouhani Hospital in Babol and then visited the two departments of chemotherapy and hematology.

The inclusion criteria comprised informed consent to enter the research, Persian language literacy, no acute physical problems, and no history of major psychiatric illnesses such as cognitive impairment, intellectual disability, and psychosis. Exclusion criteria consisted of patients with end-stage disease, who were unable to complete the questionnaire, did not answer more than 10% of the questionnaire items, were unwilling to cooperate with the researcher, and those who had a serious illness, as well as died during the research.

The researcher first explained the study to the cancer patients and invited them to participate in the study. Then, the inclusion and exclusion criteria were assessed and if the patient was eligible, the demographic information was entered into the questionnaire and filled out by the researcher. Subsequently, the questionnaires of this research were distributed among the target cancer patients after explaining the aims of the research, ensuring the confidentiality of the data, and training on the completion procedure of the questionnaire. Patients answered the questionnaires using a self- administered method and handed them over to the researcher. Enough time was given to the patients to complete the questionnaires and anonymity was ensured.

### Sample size

The sample size of the present study was set at 200 subjects based on the minimum correlation coefficient of 0.2, the 95% confidence interval, and the 80% power. Written consent forms were obtained for entry to the study.


$$n=\frac{{\left(z_{1-\alpha /2}+z_{1-\beta /2}\right)}^{2}}{w^{2}}+3$$

$$w=\frac{1}{2}\ln\frac{1+r}{1-r}$$

$$n=\frac{{\left(1/96+0.84\right)}^{2}}{{\left(0/2\right)}^{2}}+3=200$$

$$w=\frac{1}{2}\ln\frac{1+0/2}{1-0/2}=0/2$$

### Measure

Two questionnaires for evaluating spiritual well-being, anxiety, and depression were provided to patients.

### Spiritual well-being scale (SWBS)

The SWBS was designed by Ellison and Paloutzian in 1982. The 20-item SWBS was used to assess spiritual well-being. In this questionnaire, 10 questions measure religious well-being while the remaining 10 questions measure existential well-being. The spiritual well-being score is the sum of these two sub-groups, with a range of 20–120. The answers to the questions are based on the 6-point Likert scale (strongly disagree, disagree, somewhat disagree, relatively agree, agree, and strongly agree). The “strongly agree” option is assigned a score of six and the “strongly disagree” option is assigned a score of one. In negative questions, the scoring is transposed. Finally, spiritual well-being is divided into three levels: low (20–40), medium (41–99), and high (100–120). A higher score shows greater spiritual well-being [18]. The validity and reliability of this scale were evaluated by Biglari Abhari et al. (2018). Cronbach’s alpha coefficient was 0.85 [19].

### Hospital Anxiety and Depression Scale (HADS)

The HADS was developed by Herrero et al. in 2003 to assess anxiety and depression [20]. This questionnaire includes 14 questions, seven of which relate to the assessment of depression and seven of which relate to the assessment of anxiety. Overall, 21 points are obtained from each of its subscales. Its cut-off points are 0–7 for health, 8–10 for intermediate anxiety or depression disorder, and 11–21 for suspected anxiety or depression disorder for both subscales. The validity and reliability of this scale were evaluated by Kaviani et al. (2009). The Cronbach’s alpha coefficient was 0.70 for depression, and.85 for anxiety [21].

### Statistical analysis

Demographic characteristics variables were analyzed by descriptive statistics (percentage for qualitative variables and mean and standard deviation for quantitative variables). Chi-squared, T-test, and ANOVA tests were applied to determine the difference in the mean of qualitative and quantitative variables. Pearson’s correlation coefficient test was done to evaluate the correlation between the variables, and a linear regression test determined the predictors of the anxiety and depression of cancer patients. The statistical analysis was carried out using IBM SPSS version 25 software. The significance level for all tests was set at p < 0.05.

### Ethics approval

This study was confirmed by the ethics committee of Babol University of Medical Sciences with the ethical code IR.MUBABOL.HRI.REC.1398.236. The study was conducted by the Declaration of Helsinki and written consent forms were obtained from all participants prior to their entry into the study.

## Results

The mean age of the cancer patients was 51.9 ± 14.2. The highest percentage of patients was women (60.5%). The majority of participants was in the age range of 41–60 years old (46%) and had primary education (44%). The duration of cancer in the majority of patients was less than or equal to one year (74.5%). The most common type of cancer in patients was related to the breast (33.5%), followed by the gastrointestinal tract (24.5%) (Table 1).

**Table 1:** Demographic characteristics of cancer patients (N = 200).

Variable	N	%
**Gender**		
Male	79	39.5
Female	121	60.5
**Age(Years)**		
18-40	46	23
41-60	92	46
>60	62	31
**Educational Levels**		
Primary	88	44
High School	66	33
University	46	23
**Duration of illness(Years)**		
≤1	149	74.5
>1	51	25.5
**Type of cancer**		
**Breast**	67	33.5
**Gastrointestinal tract**	49	24.5
**Blood**	46	23
**Respiratory system**	16	8
**Female reproductive system**	11	5.5
**Urinary tract**	7	3.5
**Metastatic/Others**	4	2

Values are presented as the number (N) and percentage (%).

The mean scores of anxiety and depression of the cancer patients were 9.9 ± 3.7 and 9.7 ± 3.3, respectively. There was a significant difference between depression and education level (*P* < 0.001). The mean of depression was higher in people with primary education than in others. Also, there was a significant difference between age and anxiety (P < 0.02). The mean anxiety in people aged 18–40 was higher than in other age groups (Supplementary table).

The mean spiritual well-being score of cancer patients was 76.6 ± 20 (range 20–120), and in its dimensions including existential well-being 37.3 ± 9.8 and religious well-being was 39.3 ± 10.4. The highest mean dimensions of spiritual well-being were related to religious well-being in cancer patients. The majority of cancer patients had a moderate level of spiritual well-being (81%), and then high (17.5%), low (1.5%).

Table 2 shows the levels of spiritual well-being in all variables of gender, age, educational levels, duration of illness, and type of cancer were moderate. High levels of spiritual well-being were higher in men than women (21.5% vs 14.9%), in the age group above 60 years (19.4%) than 41–60 years (16.3%) and 18–40 (17.4%) years, in secondary education (28.8%) than university (19.6%), and elementary (8%). Also, patients with longer illness duration (>1 year) (17.6%), and people with gastrointestinal cancer had high levels of spiritual health (30.6%).

**Table 2:** The frequency distribution demographic characteristics and other variables based on Spiritual health levels in cancer patients (n = 200).

	Variable	Spiritual Well-being Levels					
		Low		Moderate		High	
		%	N	%	N	%	N
**Gender**	Male	2	2.5	60	76	17	21.5
	Female	1	.80	102	84.3	18	14.9
**Age(Years)**	18-40	2	4.3	36	78.3	8	17.4
	41-60	1	1.1	76	82.6	15	16.3
	>60	0	0	50	80.6	12	19.4
**Educational Levels**	Elementary	0	0	81	92	7	8
	High school	1	1.5	46	69.7	19	28.8
	University	2	4.3	35	76.1	9	19.6
**Duration of illness(Years)**	≤1 years	1	.7	122	81.9	26	17.4
	>1 years	2	4	40	78.4	9	17.6
**Type of cancer**	Breast	1	1.5	57	85.1	9	13.4
	Gastrointestinal tract	1	2	33	67.3	15	30.6
	Blood	1	2.2	37	80.4	8	17.4
	Respiratory System	0	0	16	100	0	0
	Urogenital tract, metastatic/others	0	0	19	86.4	3	13.6

Values are presented as the number (N) and percentage (%).

As shown in Table 3, there was a statistically significant relationship between educational levels and spiritual well-being (*P* < 0.049), and religious well-being (P < 0.033). People with university education had significantly higher spiritual health and religious health.

**Table 3:** The relationship between Spiritual well-being and dimensions it’s with demographic characteristics and other variables in cancer patients (n = 200).

		Spiritual Well-being		*P*-value	Religious Well-being		*P*-value	Existential Well-being		*P-* value
	Variable	M	SD		M	SD		M	SD	
**Gender**	**Male**	77.6	22.7	0.59	40.2	11.6	0.34	37.4	11.3	0.94
	**Female**	75.9	18.1		38.7	9.5		37.3	8.7	
**Age(Years)**	**18-40**	73.1	22.5	0.15	37.6	11.7	0.21	35.4	10.9	0.11
	**41-60**	75.9	18.6		38.9	9.8		36.9	8.9	
	**>60**	80.4	19.8		41.1	10.1		39.3	9.8	
**Educational Levels**	**Primary**	72.8	16.1	.049	37.1	8.3	0.03	35.7	7.9	0.09
	**High school**	79.6	22,8		40.9	11.8		38.7	11.2	
	**University**	79.8	21.5		41.1	11.2		38.6	10.5	
**Illness Duration (Years)**	≤**1 years**	77.3	19.6	0.42	39.5	10.2	0.52	37.7	9.5	0.34
	**>1 years**	74.7	21.3		38.4	11.1		36.2	10.4	
**Cancer Types**	**Breast**	76.1	17.5	0.17	38.8	9.3	0.19	37.3	8.3	0.15
	**Urogenital Tracy**	74.2	18.7		37.9	9.7		36.3	9.1	
	**Gastrointestinal tract**	82.2	24.3		42.2	12.3		40.1	12.1	
	**Respiratory System**	69.8	10.4		36.2	5.8		33.6	4.9	
	**Blood**	74.9	20.9		38.6	10.8		36.3	10.2	

Values are presented as the mean (M), standard deviation (SD), T-test, and ANOVA were done to compare the mean differences of two or more groups.

As shown in Table 4, 22% of the patients were healthy (normal) in terms of anxiety, 25% had an intermediate anxiety score, and 53% were suspected of anxiety. In healthy people, 22.7% had a moderate level of spiritual well-being and 77.3% had a high level of spiritual well-being in terms of anxiety. No person had a low level of spiritual well-being. Of people with intermediate anxiety scores, 98% had a moderate level of spiritual well-being and 2% had a high level of spiritual well-being. No person had a low level of spiritual well-being. Of people suspected of anxiety disorder, 2.8% had a low level of spiritual well-being and 63.6% had a moderate level of spiritual well-being. No person had a high level of spiritual well-being.

**Table 4:** The frequency distribution of Spiritual Well-being levels based on Anxiety/Depression levels in cancer patients (n = 200).

Variable	Spiritual Well-being Levels							
	Low		Moderate		High		Total	
	N	%	N	%	N	%	N	%
**Anxiety**							200	100
**Healthy**	0	0	10	22.7	34	77.3	44	22
**Intermediate**	0	0	49	98	1	2	50	25
**Suspected disorder**	3	2.8	103	63.6	0	0	106	53
							**Total**	
**Depression**							200	100
Healthy	0	0	11	24.4	34	75.6	45	22.5
Intermediate	0	0	68	98.6	1	1.4	69	34.5
Suspected disorder	3	3.5	83	96.5	0	0	86	43

Values are presented as the number (N) and percentage (%).

Twenty-two and a half percent of the patients were healthy in terms of depression, 34.5% had an intermediate depression score, and 43% were suspected of depression. In terms of depression, 24.4% of healthy people had a moderate level of spiritual well-being and 75.6% had a high level of spiritual well-being. No person had a low level of spiritual well-being. In people with an intermediate depression score, 98.6% had a moderate level of spiritual well-being and 1.4% had a high level of spiritual well-being. No person had a low level of spiritual well-being. Of people suspected of depression, 3.5% had a low level of spiritual well-being and 96.5% had a moderate level of spiritual well-being. No person had a high level of spiritual well-being.

The findings of this analysis have shown that anxious people (intermediate and suspected of anxiety disorder) compared to healthy people had lower mean spiritual well-being, religious Well-being, and existential Well-being. Pearson’s correlation coefficient matrix revealed a significant negative correlation between religious Well-being (rho = -0.832, *P* < 0.001) and existential Well-being (r = -0.830, *P* < 0.001) with anxiety. Furthermore, depressed people (intermediate and suspected of depression disorder) compared to healthy people had lower mean spiritual health, religious Well-being, and existential Well-being. There was a significant negative relationship between religious well-being (rho = -0.842, *P* < 0.001) and existential well-being (r = -0.813, *P* < 0.001) with depression. In other words, with an increase in religious well-being or existential well-being, anxiety and depression in cancer patients decreased (Table 5).

**Table 5:** Spiritual Well-being and its dimensions based on anxiety and depression (n = 200).

Variable	Anxiety		
	Healthy	Intermediate	Suspected disorder
	M(SD)	M(SD)	M(SD)
**Spiritual Well-being**	105.8 (13.7)	105.8 (13.7)	63.4 (8.9)
**Religious Well-being**	54.3 (7.2)	54.3 (7.2)	32.5 (4.8)
**Existential Well-being**	51.5 (6.7)	51.5 (6.7)	30.9 (4.5)
	**Depression**		
**Spiritual Well-being**	105.8 (14.5)	75.4 (10.1)	62.3 (9.2)
**Religious Well-being**	54.5 (7.3)	38.7 (5.1)	31.7 (4.7)
**Existential Well-being**	51.3 (7.5)	36.7 (5.3)	30.6 (4.8)

Values are presented as the mean (M), standard deviation (SD).

There was a significant positive relationship between anxiety and depression (rho = 0.717, *P* < 0.001). In other words, as the level of anxiety increased, the level of depression also increased.

The Linear Regression model method was used to investigate the predictive effect of variables on the main variable of anxiety and depression. In this method, in the independent classified variables, the first category was defined as the reference category, and the total score of anxiety, depression, and spiritual well-being were entered as independent variables in the model. According to Table 6, the variables of spiritual well-being and age could significantly and negatively predict anxiety. In other words, with the increase in spiritual well-being and age, the level of anxiety in cancer patients decreased. Based on the results of the table, for each score of increase in spiritual well-being, the anxiety score was reduced by 0.154 and for each year of age increase, the anxiety score was reduced by 0.3. Furthermore, spiritual well-being and education levels were negative predictors of depression. In other words, with the increase in spiritual well-being and also education levels of cancer patients, the risk of depression decreased. For each score of increase in spiritual well-being, the score of depression decreased by 0.134. Also, the level of middle/high school education compared to Primary school 0.885, and university education compared to Primary school 1.66 reduced the depression score.

**Table 6:** Results of regression model for anxiety and depression with the spiritual Well-being, and other variables in cancer patients (n = 200).

Independent Variable	t	SE	Beta	*P* Value	t	SE	Beta	*P* Value
**Anxiety**					**Depression**			
**Spiritual Well-being**	-20.5	.008	-.15	.001	-21.5	.006	-.13	.001
**Age(Years)**	-2.4	.01	-.30	.017	1.4	.01	.01	.15
**Gender**								
Male(R)								
Female	.67	.38	.256	.50	1.5	.31	.49	.12
**Educational levels**								
Primary(R)								
Middle/high school	-.20	.36	-.723	.84	-2.9	.30	-.88	.004
University	-1.6	.41	-.643	.12	-4.8	.34	-1.6	.001
**Illness Duration (Years)**								
≤1 years(R)								
>1 years	-.48	.34	-.164	.63	.54	.28	.15	.59
**Cancer Type**								
Breast(R)								
Urogenital Tract	1.0	.52	.527	.31	-.98	.43	-.42	.33
Gastrointestinal tract	.33	.47	.157	.74	.04	.39	.01	.97
Respiratory System	-.03	.67	-.170	.98	.27	.60	.01	.79
Blood	-1.3	.44	-.584	.18	-1.2	.36	-.44	.22

A Linear regression test was done to determine the predictors of anxiety and depression.

## Discussion

The purpose of this research is to investigate whether diverse spiritual well-being approaches have a role in supporting psychological activity engagement in women and men with cancer who were referred to Ayatollah Rouhani Hospital in Babol.

In this study, nearly half of cancer patients had anxiety and depression disorders, which was in line with the study of Grassi et al. (2023). There are consistent reports of a high level of mental health problems such as depression in cancer patients [7]. Esfahani et al. (2020) reported that one-fifth of Iranian women with breast cancer were depressive [22]. In this regard, Safaie et al. (2022) reported that definitive diagnosis, boring treatment process, and long treatment duration can lead to psychiatric disorders in cancerous patients, such as anxiety and depression [23]. Therefore, the care programs can be a good strategy to decrease the anxiety and depression of cancerous patients.

The gathered data revealed that with the increase in age, the level of anxiety in cancer patients decreased. In other words, age was a significant negative predictor for anxiety. The mean anxiety in people aged 18–40 was higher than in other age groups. A review of the literature revealed a negative significant relationship between ages with anxiety [17]. The anxiety and depression disorders were less prevalent in older than younger adults [24]. In explaining the above results, it can be stated that increasing age can be accompanied by increasing experiences and ability to regulate emotional responses to stress. This may be because of age-related improvements in experience as well as emotion regulation, which may lead to the correct management of anxiety in difficult situations [25]. Although, factors contributing to this lower prevalence of anxiety need further investigation.

The result found that educational level was a significant and negative predictor for depression. With the increase in education levels, the risk of depression decreased in cancer patients. In other words, people with primary education had significantly more depression than with those higher education, which was in line with the study of Khezri et al.(2015) [17]. It seems that patients with higher educational levels have more information related to cancer and treatment methods, which can decrease mental disorders including depression.

The results of the present study showed that the majority of cancer patients had moderate spiritual well-being. Also, the results showed that the mean score of religious well-being was higher compared to existential well-being in patients. In Hedayatizadeh Omran et al. (2018) study on cancer patients undergoing chemotherapy, the mean score of spiritual well-being was reported as moderate level [26]. In addition, Musarezaei et al. (2012) indicated moderate levels of spiritual well-being and also more religious well-being compared to existential well-being in women with breast cancer. Therefore, paying attention to the spiritual well-being of these patients along with the physical aspects of the treatment process by the medical staff will help improve the mental health of the patients [27]. Considering the religious context of our society, the above results were predictable.

The result of the present study showed that cancerous patients with a university education had significantly higher spiritual well-being and also religious well-being. A review of the literature showed a significant association between religious health and educational level [28].

According to the findings of this study, the variables of spiritual well-being could significantly and negatively predict anxiety. Furthermore, there was a significant negative relationship between religious and existential Well-being with anxiety. Anxious people (intermediate and suspected of anxiety disorder) compared to healthy people had lower mean spiritual, religious, and existential Well-being. These findings were consistent with the study of Madadi Ardakani et al. (2019) [29], Khademvatani et al. (2015) [28], and Khezri et al. (2015) in Iran [17]. Also, other studies found a significant inverse relationship between spirituality dimensions and death anxiety in cancer patients undergoing chemotherapy. Furthermore, the component of spiritual beliefs in life was a suitable predictor for reducing the death anxiety of cancer patients; death anxiety was predictable with religious prejudice [26,30]. A review of the literature revealed religious comfort was inversely related to anxiety, so that women with cancer who received relief from the feeling of spirituality had lower anxiety [31]. Spiritual well-being refers to a set of abilities, capacities, and spiritual resources whose use increases the adaptability and, consequently, the mental health of persons [32]. It is one of the main elements of enjoying health [16], which coordinates the relationship between internal forces, and leads to stability of life, peace, symmetry and harmony, emotions, and having a close relationship with God [33]. Spiritual well-being is one of the basic concepts regarding how to deal with the problems [11]. It appears that a comprehensive care program that includes spiritual well-being may be effective in reducing mental problems among cancer patients [29]. In explaining the above results, it can be stated that spirituality can also vary from culture, religion and persons. The discussion will be richer if the author compared among different cultures. Spirituality, religion, and culture are intertwined and have a strong impact on each other [34], which needs further research on this topic.

The gathering data of the present study revealed that spiritual well-being was a negative predictor of depression. In other words, with the increase in spiritual well-being, the level of depression decreased in cancer patients. Furthermore, there was a significant negative relationship between religious and existential Well-being with depression. Depressed people (intermediate and suspected of depression disorder) had lower religious and existential well-being compared to healthy people, which was in line with the study of Musarezaei et al. (2012)[27] and Khademvatani et al. (2015)([28]. Pasha et al. (2023) revealed a significant negative relationship between spiritual well-being and its dimensions with mental health [35]. The use of religious and spiritual beliefs can be considered a useful and constructive coping strategy for promoting psychological health [36]. The researchers reveal that increasing the level of meaning in life and spirituality not only helps to overcome inconsistencies but also increases life satisfaction [33]. Spirituality is often considered a constructive coping strategy for improving the mental health of individuals, and emphasized by psychologists in the past few decades. Spiritual well-being harmonizes the dimensions of a person’s health, which enhances their adaptability and mental function [11]. This research provides evidence of the important protective function of spiritual well-being and its unique role in maintaining mental health [37,38], which poses significant anxiety to individuals, particularly cancer patients who are more vulnerable to anxiety. It seems that spiritual well-being and its dimensions can be attributed to the effect of communication and trust with God, and the resulting more peace and hope in cancer patients. Therefore, religious beliefs can be used to improve the health of patients, and resolve depression [39].

Our findings showed a significant positive relationship between anxiety and depression. Cancer patients with depressive disorder were more anxious, which was in line with Vowels et al. (2022) [40], and Shek et al. (2022) [41] studies. Anxiety significantly predicts depression; a drop in anxiety predicts a drop in depressive symptoms. There is also an intimate link between depression and other negative emotional states, particularly anxiety. Another study indicates the coexistence of anxiety and depression. In other words, depression contributes to anxiety; anxiety results in depression [42]. Persons with high anxiety have low attachment security, low instrumental coping skills, and elevated affiliative focus, which are disposed to depression when coping with “Unpleasant life events” [43].

## Limitations

The findings of this study cannot be generalized to the entire cancer patients. Furthermore, there was cultural diversity in the Iranian context in the study, as well as spirituality can vary in culture, religion and persons, which needs further research. Another limitation of the present research may be the lack of accuracy when completing the questionnaire because of dire cancer-related conditions and treatment procedures.

## Conclusion

Our study results revealed that spiritual health can play a positive role in reducing anxiety and depression in cancer patients and promoting mental health, which may help in the recovery from the disease. Therefore, clinical monitoring and considering spiritual health as part of the treatment approaches for cancer patients are an important need.

### Informed consent

All cancer patients were requested to complete informed consent before starting the study.

## Supporting information

S1 Table Anxiety/Depression means’ based on demographic characteristics and other variables in the cancer patients (n = 200). (DOCX)
