# Supplementary material for: Relationship between spiritual well-being with anxiety and depression among cancer patients
Source: PLoS One. 2025 Jun 18;20(6):e0322923. doi: 10.1371/journal.pone.0322923 (PMC12176229; doi:10.1371/journal.pone.0322923)
Supplement: S1 Table — (DOCX) [file pone.0322923.s001.docx]

**Table S1.** Anxiety/Depression means' based on demographic characteristics and other variables in the cancer patients (n=200)

| ***P*-value** | **Depression** | | ***P*-value** | **Anxiety** | | **Variable** | |
| --- | --- | --- | --- | --- | --- | --- | --- |
|  | **SD** | **M** |  | **SD** | **M** |  |  |
| 0.20 | 3.7 | 93 | 0.17 | 4.1 | 9.5 | **Male** | **Gender** |
|  | 3.1 | 9.9 |  | 3.5 | 10.3 | **Female** |  |
| 0.90 | 3.8 | 9.5 | 0.02 | 4.3 | 11.2 | **18-40** | **Age(Years)** |
|  | 3.1 | 9.7 |  | 3.5 | 9.9 | **41-60** |  |
|  | 3.3 | 9.8 |  | 3.5 | 9.2 | **>60** |  |
| 0.001 | 2.7 | 10.9 | 0.93 | 3.1 | 10.6 | **Primary** | **Educational Levels** |
|  | 3.3 | 8.9 |  | 4.1 | 9.8 | **High school** |  |
|  | 3.6 | 8.3 |  | 4.1 | 9.2 | **University** |  |
| 0.52 | 3.2 | 9.6 | 0.83 | 3.8 | 9.9 | ≤**1 years** | **Illness Duration (Years)** |
|  | 3.7 | 9.9 |  | 3.6 | 10.1 | **>1 years** |  |
| 0.27 | 2.9 | 9.9 | 0.19 | 3.2 | 10.3 | **Breast** | **Cancer Types** |
|  | 2.4 | 10.1 |  | 3.1 | 11 | **Urogenital Tract** |  |
|  | 3.8 | 8.9 |  | 4.7 | 9 | **Gastrointestinal tract** |  |
|  | 2.2 | 11 |  | 2.4 | 10.7 | **Respiratory System** |  |
|  | 3.9 | 9.4 |  | 3.9 | 9.8 | **Blood Circulation** |  |

Values are presented as the mean (M), standard deviation (SD), T-test, and ANOVA were done to compare the mean differences of two or more groups.
